# Biomechanics in Soft Mechanical Sensing: From Natural Case Studies to the Artificial World

**DOI:** 10.3390/biomimetics3040032

**Published:** 2018-10-24

**Authors:** Afroditi Astreinidi Blandin, Irene Bernardeschi, Lucia Beccai

**Affiliations:** 1 Center for Micro-BioRobotics, Istituto Italiano di Tecnologia, Pontedera, 56025 Pisa, Italy; irene.bernardeschi@iit.it; 2 The BioRobotics Institute, Scuola Superiore Sant’Anna, Pontedera, 56025 Pisa, Italy

**Keywords:** mechanical sensing, tactile sensing, flow sensing, mechanoreceptor, bioinspired sensing, biomimetics, soft robotics, soft sensors, morphological computation, biomechanics

## Abstract

Living beings use mechanical interaction with the environment to gather essential cues for implementing necessary movements and actions. This process is mediated by biomechanics, primarily of the sensory structures, meaning that, at first, mechanical stimuli are morphologically computed. In the present paper, we select and review cases of specialized sensory organs for mechanical sensing—from both the animal and plant kingdoms—that distribute their intelligence in both structure and materials. A focus is set on biomechanical aspects, such as morphology and material characteristics of the selected sensory organs, and on how their sensing function is affected by them in natural environments. In this route, examples of artificial sensors that implement these principles are provided, and/or ways in which they can be translated artificially are suggested. Following a biomimetic approach, our aim is to make a step towards creating a toolbox with general tailoring principles, based on mechanical aspects tuned repeatedly in nature, such as orientation, shape, distribution, materials, and micromechanics. These should be used for a future methodical design of novel soft sensing systems for soft robotics.

## 1. Introduction

**Figure 1:**
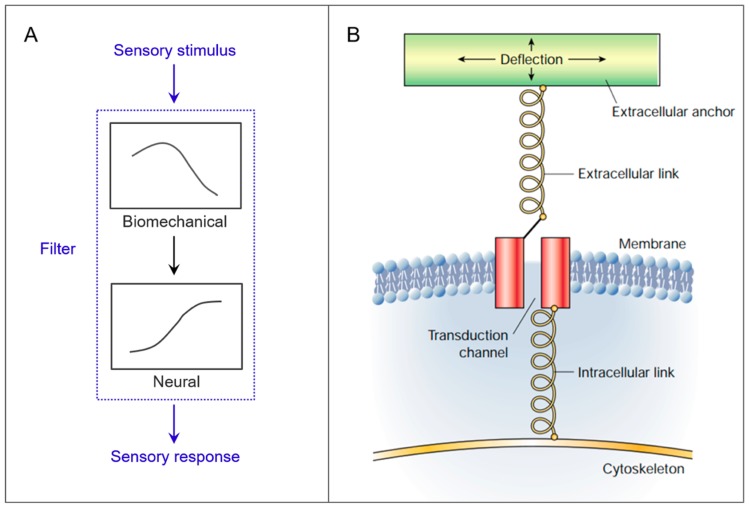
General features of mechanical sensing in nature. (**A**) The outer stimuli are filtered both biomechanically and neurally, before producing sensory responses in the living organism (adapted from [1] by permission of Oxford University Press). (**B**) General features of mechanosensory transduction at the cellular level (reprinted by permission from Springer, [8]). Transduction of the stimuli occurs after deformations and/or displacements of structures.

Mechanical sensing, or mechanosensing, is vital in living beings and robots in order for them to interact with the environment, as well as to implement movement and action. It involves the detection and transmission of information about various types of cues (e.g., contact, pressure, force, strain, flow, vibration, acceleration, and directionality). This information may be used for the perception of stimuli from the environment (exteroception) or the self (proprioception). The appropriate design of a natural sensory organ results in its ability to filter the input signal [1] (Figure 1A) and reduce computation needs [2]. The biomechanical part of the filter can partly compensate for the neural part, since they have “mutually exclusive roles” [1]. In robotics, intelligent biomechanical design results from an effective use of embodiment, in other words from adapted morphology and materials [3]. As a consequence, through morphological computation, defined as computation obtained through interactions of the physical body with the environment and/or itself, it becomes possible for the structure to eventually “subsume part of the role of the controller” [2,4]. Specifically for sensing, morphological computation facilitates perception by preprocessing sensory information [5]. Examples about current artificial sensing systems taking advantage of morphological computation can be found in Bernth et al. [6]. Both concepts of embodiment and morphological computation are fundamental in robotic applications and in particular in the case of gathering information from the surrounding environment through the sense of touch. Indeed, morphological computation facilitates perception [5], not only when the sensory data are acquired through active movement and selected to refine movements, that is, active touch [5,7], but also when acquired through passive interaction with the environment.

In nature, forces are not sensed directly. Mechanical stimuli induce deformations and/or displacements of tissues and specialized sensory organs, which are soft either through materials themselves, or through deformable architectures. Stimuli are only later detected and transduced in electrochemical signals through the mechanotransduction process [8,9] (Figure 1B). Hence, the mechanical behavior of a sensing structure itself is of particular importance, as the electrochemical transduction of the signal. This concept can be the key for creating new sensing strategies in soft robotics [10], where complex computation must be avoided, and making use of morphological computation is particularly critical.

In this study, our attempt is to report and discuss cases of sensory systems that distribute their intelligence in both structure and materials. By doing this, we aim at extracting and analyzing specific principles for mechanical sensing from natural cases and at highlighting their existent and/or possible role for the design of novel artificial sensing systems. A number of examples from the animal and the plant kingdoms are detailed. A focus is set on biomechanical aspects, such as morphology and material characteristics of the selected sensory organs and their environment, and on how their function is affected by them. Covering a large range of sensory organs of different morphologies allows us to examine the possibility of principles being shared and consequently generalized. On the other hand, examples of artificial sensors that implement these principles are provided and/or ways in which they can be translated artificially are suggested.

## 2. Case Studies

**Figure 2:**
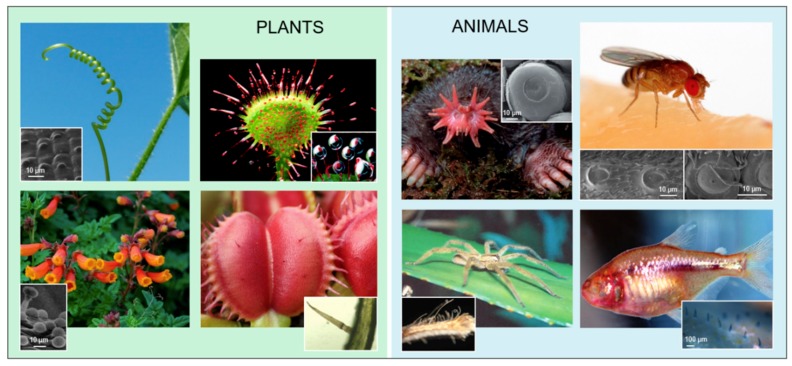
Overview of examples of specialized mechanical sensory organs with significant biomechanical aspects in nature. The different sensor type morphologies are encountered in the plant and animal kingdoms. **Plants**: (top left) *Bryonia dioica* Jacq. tendril (A. Moro, Department of Life Sciences, University of Trieste, CC BY-SA 4.0 [37]), (inset) tactile bleps on tendril (reprinted by permission from Springer, [30]); (top right) *Drosera rotundifolia*, (inset) close up of tentacle tips (photos by Barry Rice, http://www.sarracenia.com); (bottom left) *Eccremocarpus scaber* (CC BY-NC-ND 2.0 [38]), (inset) tactile papillae on tendril (reprinted from [32] by permission of John Wiley & Sons, Inc.); (bottom right) *Dionaea muscipula* (reproduced with permission from FlyTrapCare.com), (inset) trigger hair (photo by Martin Brunner, CC BY-SA 2.5 [39]). **Animals**: (top left) star-nosed mole, (inset) Eimer’s organ on star (adapted from [29], Copyright 2012, with permission from Elsevier); (top right) *Drosophila melanogaster* (photo by Sanjay Acharya, CC BY-SA 4.0 [37]), (inset) campaniform sensilla on halteres (adapted from [33], Copyright 2017, with permission from Elsevier); (bottom left) *Cupiennius salei* (reprinted by permission from Springer, [34]), (inset) spider hair sensilla (adapted from [35], Copyright 2004, with permission from Elsevier); (bottom right) *Astyanax fasciatus*, (inset) cupulae of neuromasts on the lateral line (reproduced from [36] with permission from The Royal Society of Chemistry).

In this work, we focus on a selection of salient cases of specialized mechanical sensory organs of animals, including insects and other arthropods, along with plants, described from a biomechanical point of view. Out of all soft mechanical sensing structures available in nature, a well-known example of a mechanical sensory organ is the human skin [11,12,13,14,15], from which many artificial sensing technologies have already been inspired [16,17]. The absence of neurons in plants and the relatively simple [18] role of the neural filter in arthropods might give a greater importance to their biomechanical filters. Specialized sensory organs from the plant and animal kingdoms are organized here according to the most frequent morphological traits—cantilevers, cantilevers with domes, and domes. Details about different cases are reported in Table 1, while some examples are depicted in Figure 2 [19,20,21,22,23,24,25,26,27,28,29,30,31,32,33,34,35,36]. Out of these cases, most structures are found at the microscale and clustered in groups. Whereas domes seem to be more present in air and cantilevers with domes in water, cantilevers are found in both environments. While their function varies, cantilevers with domes are reported to sense flow.

### 2.1. Cantilevers

Cantilevers or hairs are the first category of mechanical sensory organs studied here. Hairs serve functions of insulation and particle filtering, but also multiple sensing purposes, such as flow, balance, inertia, touch, and chemical and temperature sensing [52]. Located at joints, hair structures can also function as proprioceptors [53,54]. The variation of their structural features contributes to enabling functions [55]. Since the spider *Cupiennius salei* and its mechanosensory hairs or sensilla have been extensively studied, they are included here along with vibrissae, which have been frequently used in robotics.

#### 2.1.1. Arthropod Sensilla

**Figure 3:**
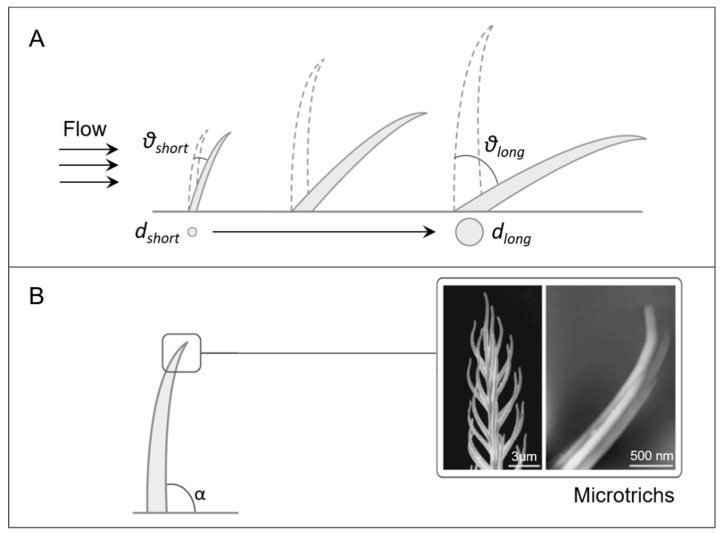
Biomechanical principles of spider sensilla for air flow sensing (trichobothria). (**A**) The length of trichobothria varies, among the same group and also in different media, following the variation of the boundary layer thickness. Different lengths correspond to different best frequencies’ sensitivity. In longer hairs, the diameter *d* of and the deflection angle *θ* are larger than in shorter hairs. (**B**) Hairs present an angle *α* of about 90° with respect to the body and microtrichs that might increase sensitivity at low airflows (adapted from [58] with permission from The Royal Society).

**Figure 4:**
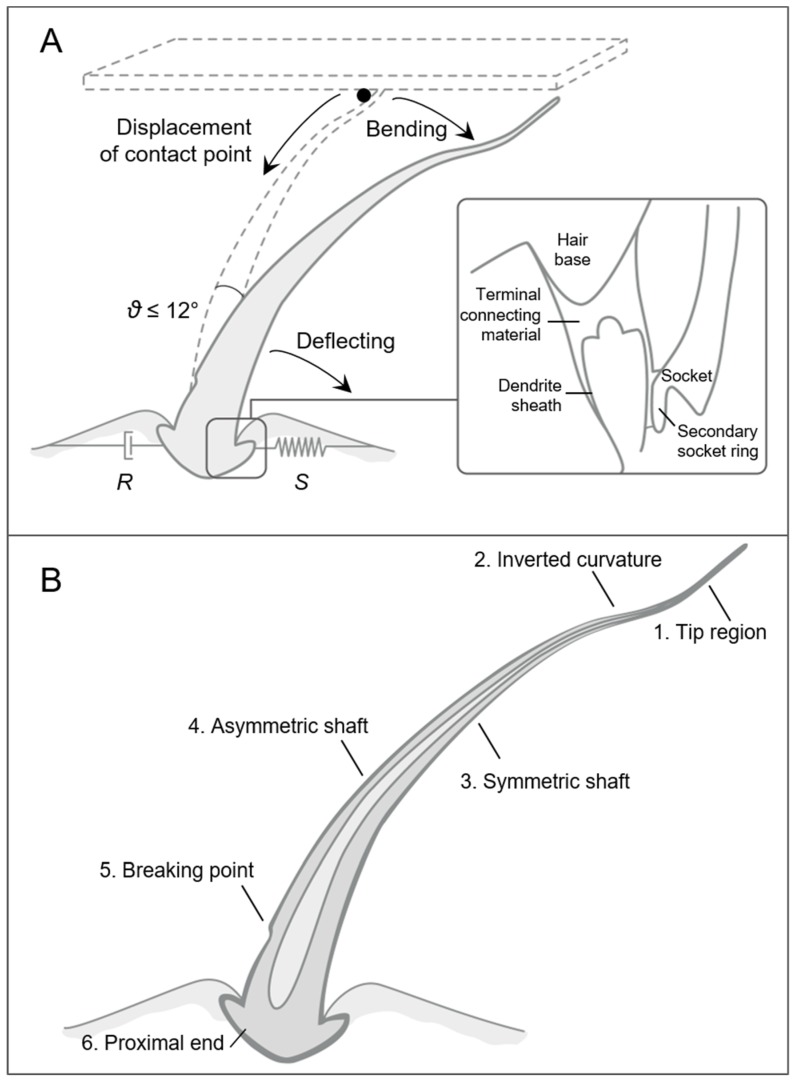
Biomechanical principles of spider tactile hairs in tactile sensing. (**A**) Bending in addition to deflection protects the hair against breaking. The maximum deflection angle of the hair *θ* is 12°, with a damping element *R* and a spring element *S*. (Inset) The hair base is finely tuned, with the presence of a “terminal connecting material” and a “second joint”, described in the text (reprinted by permission from Springer, [81]). (**B**) Regional heterogeneity along the hair attributes different functions and optimization of each part (reprinted by permission from Springer, [56]). The regions depicted have the following characteristics: (1) plastic region, abrupt decrease of lumen diameter, strong curvature in different directions; (2) approximately one third of hair length, not found in all hairs; (3) rotational symmetry; (4) strong deflections, wall of the hair thicker towards the tarsus; (5) decrease of outer diameter towards the base; (6) morphologically and functionally most complex structure of the hair.

Among sensory organs, arthropod sensilla constitute one of the most studied groups, especially on *C. salei*. Related studies include finite element method (FEM) modeling [56], electrophysiological observations [57], and the establishment of mathematical models for the characterization of their behavior [58]. Most hairs serve a mechanosensory function, while some function as chemoreceptors [45]. Mechanosensory sensilla of arthropods can be subdivided into tactile hairs and trichobothria, which function as medium flow sensors [45] (Figure 3 and Figure 4). Both types of sensilla are located within a cuticular socket, where they are coupled with dendrites. The mechanical behavior of this viscoelastic suspension can be modeled as a spring element *S* applying a torsional restoring force and a damping element *R* [35,58].

##### Trichobothria

About 100 trichobothria can be found on each leg of *C. salei*, mostly dorsally, typically in groups of 2–30 hairs [59]. Through their length, they are mechanically tuned to different frequency ranges between 40 and 600 Hz [60]. The threshold stimulus lies at airflows as low as 0.15 mm/s [61]. In the range of about 50–100 Hz, frequencies are detectable at deflection angles as small as 0.01–0.1°, while frequencies from 10 to 500 Hz are detectable at a higher threshold (i.e., deflection angles of 1°) [54,60].

The biomechanical principles used by trichobothria in mechanosensing and possibly in artificial sensors are detailed here and illustrated in Figure 3. The length of trichobothria within groups increases from proximal to distal, ranging from 0.1 to 1.4 mm for the different leg parts [59] (Figure 3A). Interestingly, different lengths correspond to different best frequencies’ sensitivity, the shorter ones being most sensitive in the high-frequency range [60]. Furthermore, these lengths correspond to the range of the biologically relevant boundary layer thicknesses [62]. In addition, the length ranges correspond to various types of terrestrial arthropods [35]. The thickness of the boundary layer created varies according to the medium. Indeed, typical mechanosensing hairs in water seem to be shorter than in air, due to smaller boundary layers in water [62]. Regarding the diameter, it is 5–15 µm at the base while smaller at the hair tip, and long hairs are thicker than short ones [59]. The influence of diameter is predicted to be minor in water, unlike in air [62]. These findings highlight the importance of the environment where the sensory organ is intended to be used.

During stimulation, trichobothria are considered to be only subjected to deflection because of the small values of spring stiffness and inertial resistance, in the order of 10^−12^ Nm/rad and 10^−14^–10^−15^ Nm/rad, respectively [58,59]. These extremely small constants cause the hair shaft to tilt without bending when driven by the viscous force of air [34]. They are therefore considered as stiff rods with a flexible pivot [35]. Deflections are higher for hairs that are longer, within the same group (Figure 3A), and also for groups located more distally [59]. Moreover, in order to understand the influence of density, fluid dynamics studies for the hair-to-hair interaction have been developed, in particular viscous coupling [63,64,65]. This effect is considered to be negligible for trichobothria, due to relatively large spacing [63]. 

Finally, a notable feature of trichobothria is the presence of microtrichs with striations on their surface [58,66] (Figure 3B). Although, to the authors’ knowledge, the function of microtrichs has not been defined, an hypothesis is that they augment sensitivity at low airflows, also supported in [59,67]. Under such conditions, the thickness of the boundary layers around single microtrichs augments, leading to an increased average airflow resistance and causing it to function as a paddle rather than as a rake. They also contribute to a lightweight design [59].

**Figure 5:**
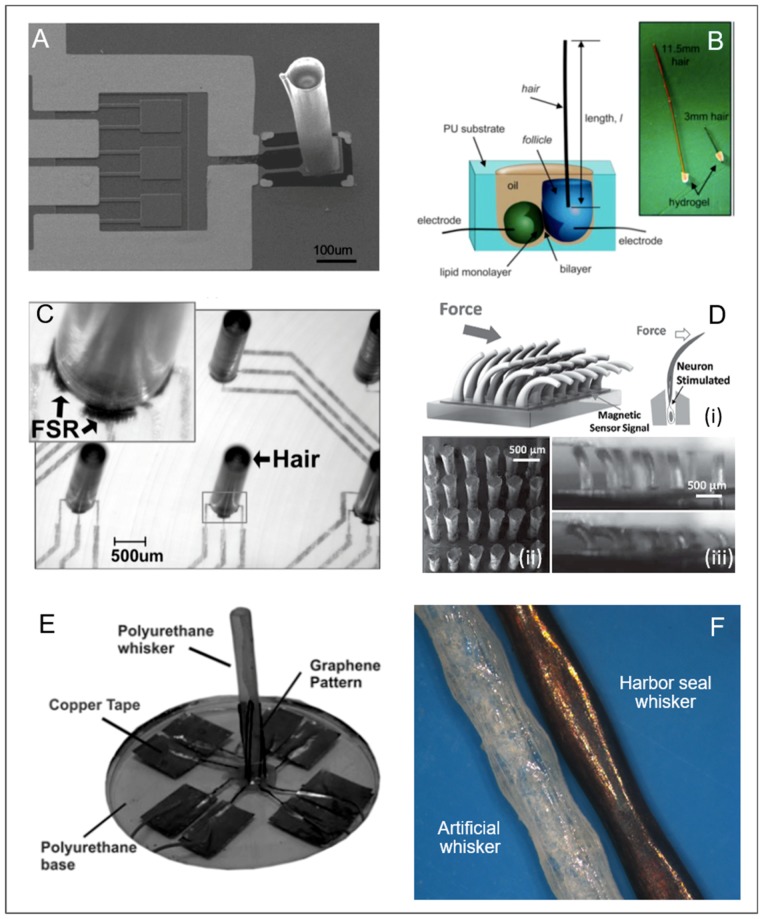
Artificial sensors integrating cantilever shapes. (**A**) Stiff SU-8 hair sensor (© 2007 IEEE. Reprinted, with permission, from [71]). (**B**) Synthetic fiber in a hydrogel follicle, using a lipid bilayer as the transduction element (reproduced from [76] with permission from The Royal Society of Chemistry). (**C**) Polyurethane force sensitive resistor (FSR) allowing bending (© 2006 IEEE. Reprinted, with permission, from [83]). (**D**) Sensor with array of cilia using magnetoimpedance (reprinted from [84] by permission of John Wiley & Sons, Inc.). (**E**) Artificial whisker with straight cylindrical shape (reproduced from [108]. The publisher for this copyrighted material is Mary Ann Liebert, Inc. publishers). (**F**) Artificial whisker with undulated surface compared to a harbor seal whisker (republished with permission of Annual Reviews, from [49]).

Regarding artificial stiff hairs for flow sensing, various sensors have been developed [68], in particular exploiting deflection in association with different composition materials and transducing principles [69] (Figure 5). Sensors with cylindrical stiff SU-8 polymer hairs have been accounted for good performances, some reaching low thresholds of less than 1 mm/s in air [70] or water [71] (Figure 5A), which is high compared to the threshold in trichobothria (0.15 mm/s) [60]. As Liu mentions, extraction of biomimetic principles will provide solutions to engineering challenges [72]. An example is the artificial beam developed, whose cuboid shape with unequal adjacent faces favors its deflection in the orientation of the broad face, while a cylindrical/pyramidal cantilever would deflect following stimuli from all directions [72]. Further cases of SU-8 hairs involve either a capacitive working principle with a flexible copper top electrode attached to the hair base, having a flexible electrode gap of air or water [73] (Figure 5B), or a piezoresistive working principle with a compliant rubbery base [74]. In both of them, a resemblance with the trichobothrium pivot can be observed. An additional future development that was suggested by McConney et al. [58] is the use of viscoelastic material instead of silicon substrate, mimicking the viscoelasticity of the biological hair suspension. This material would act soft enough to transmit mechanical energy, but at low frequencies with respect to the frequency of interest, mechanical energy would be lost through the hair, while at frequencies above the acceptable range the base would become rigid with a consequent inefficient strain of the piezoresistive component [74]. Metal liquid alloy has also been proposed as piezoresistor, with a polydimethylsiloxane (PDMS) membrane used as support [75]. Other interesting artificial flow sensing approaches take inspiration from hair cells, by embedding the hair base in a soft support and using alternative biomimetic mechanotransduction principles [76,77].

Another aspect that could be exploited in artificial devices is the variation of boundary layer thicknesses, like in the biological model. In new artificial designs, it could be useful to define lengths of hairs according to types, frequencies, and velocities of flows expected, and medium used. In air, varying the diameter of the structure could also tune to the desired function. Designing arrays with a heterogeneous distribution of hair directions (angle between the hair and the flow) could enhance sensitivity as suggested by data collected by Steinmann et al. [78]. Finally, microtrichs can be fabricated in order to exploit their function. The size and number of microtrichs or corresponding flow resistance structures can be augmented, if higher sensitivity to low flows is required.

##### Tactile Hairs

Tactile hairs on *C. salei* have a density of up to 400 per mm^2^ [35], while on human fingertips the density of the mechanoreceptors Merkel cells and Meissner’s corpuscles is barely about 70 and 140 per cm^2^, respectively [79,80]. Their length reaches 3.2 mm (metatarsus) and 2.6 mm (tarsus), while the diameter of the hair shaft base is 23 and 20 µm, respectively [57]. In spite of their multiple innervation, tactile hairs do not seem to be provided with additional information about directionality [57]. The displacement closest to the dendrites is of 0.05 µm (at a threshold stimulus of 1°) [81]. 

The biomechanical principles used by tactile hairs in mechanosensing and possibly in artificial sensors are detailed here and illustrated in Figure 4. Spring stiffness in tactile hairs’ articulation is about four powers of ten higher than in trichobothria (in the order of 10^−8^ Nm/rad) [57], forcing the hair shaft to bend in addition to deflecting [45]. One of the consequences is that the base is not deflected by more than 12°, protecting the hair against breaking [35] and augmenting the working range. The forces needed to deflect the hair range between 10^−5^ and 10^−4^ N/° [61]. The protection effect is also generated through the reduction of the effective lever arm following the displacement of the contact point towards the hair base when augmenting stimulus intensity [56] (Figure 4A).

Another characteristic of tactile hairs is their micromechanical regional heterogeneity in their cross-sections, considering hair diameter, wall thickness, and curvature, which gives them a slight S-shape (Figure 4B). The symmetry of the shaft wall thickness in the central region of the hair might be related to the multiple directionality of the stimuli, while the asymmetry of the shaft wall thickness close to the base, where maximal stresses occur, functions as a measure against buckling. Such a design gives a lightweight structure while saving material [56].

Finally, the mechanics of the hair base are of key importance. The presence of a “second joint” [45] in the hair’s socket makes the hair bend before contacting the socket [81] (Figure 4A, inset). Notably, the coupling of the hair base and the dendrite occurs indirectly, through the “terminal connecting material”, whose deformation is thought to protect dendrites by absorbing most forces exerted [81]. The coupling is not direct, unlike for insects [82].

Adopting bending in addition to deflecting in artificial sensors—for example, by using an elastomeric hair material—could reduce their probabilities of breaking. The sensors by Engel et al. [83], with a polyurethane hair on top of four force sensitive resistors, are more robust compared to silicon pillars, since they can be bent by 90° (Figure 5C). Moreover, this tactile sensor displays a 0.1° detection threshold, compared to the 1° detection threshold in tactile hairs of *C. salei* [83]. However, comparing the forces needed to deflect the cantilevers, they reach 25 × 10^−5^ N/° for the artificial cantilever, while barely 5 × 10^−5^ N/° are needed for the biological one [61]. It should be kept into account that measurement methods for both studies should be equivalent. This example reinforces our initial hypothesis that, while major force sensing solutions have been implemented by optimizing the computational part of the filter (Figure 1A), improvements on the aspects like the detection threshold could still be achievable by optimizing the mechanical transmission of the stimulus. In another work, sensors were made multimodal, sensing texture and normal forces, through bending of PDMS pillars with embedded iron nanowires, detected by a giant magnetoimpedance sensor [84] (Figure 5D). Bending of cantilever structures with detection by strain gauges has also been used for surface and flow sensing [85]. 

However, specialized artificial designs with mechanical requirements of regional heterogeneity along the hair can be tested. For example, in regions subjected to maximal stress, use of thicker wall on the wall opposite to stimulation could prevent breaking, and accordingly symmetric wall thickness in parts with equal probabilities of direct contact with the stimulus. Like for the biological structure, developing complex mechanisms for the hair base of the artificial sensor could have a drastic influence on its mechanical behavior. Following the “second joint” approach described earlier, having an internal socket in the hair base would contribute to hair bending. If necessary, adding a “terminal connecting material” in the socket would protect sensitive inner components by absorbing forces. Finally, since no hysteresis was noticed when stimulating tactile hairs [56], investigating and exploiting this property could give indications on building hair-like tactile sensors.

#### 2.1.2. Vibrissae

Vibrissae, or otherwise whiskers when located on the face, might be the most characteristic sensing hair structure that comes to our minds. Indeed, they are found in numerous mammals, such as rodents, and marine mammals, such as seals, manatees, or sea lions. In addition to enabling touch, vibrissae also detect hydrodynamic [86,87] and presumably airflow stimuli [88], and even proprioception. Notably, vibrissae are used for the implementation of active touch. This involves the active movement of the sensory organs by the animal—for example, modifying the organ orientation to the stimulus for the tactile exploration of the environment. This way, animals can discriminate location [89], size [90], shape [91], orientation [92], or roughness [93,94].

Since active touch is important in robotics, various artificial whisker systems have been developed, including cases inspired from the natural ones [95]. While initial active sensors were used for obstacle avoidance [95], recent artificial active whiskers may also be used for surface inspection, by detecting, for instance, whisker deflection torque [96], force and position through quartz resonance [97], or strain [85].

In nature, mechanical properties presented might vary, not only across species, but also across the same region of vibrissae [98]. Whiskers are found in a shape of tapered and intrinsic curvature [99]. When encountering an object and undergoing a collision, vibrissae mostly move in the direction of the concave side, maintaining the contact with the object longer and with a higher force [100]. The surface is another morphological aspect that varies among whiskers. The undulated surface of harbor seal vibrissae suppresses vortex-induced vibrations [101,102]. This effect helps the seal to keep the vibrissae still while swimming, while in slowly moving water the sea lion with the smooth vibrissae surface displays a higher sensitivity [103]. 

The elasticity of the beam influences the deflection and therefore the sensitivity directly, because of the dependence of the bending stiffness *K* on the Young’s modulus *E* and the second moment of area *I*, given from the equations K=EI, where in the case of the beam $I=\frac{{\pi r}^{4}}{2}$ [104]. Vibrissae are characterized as stiff, having a high Young’s modulus, which varies along its major axis. While the average Young’s modulus of a rat vibrissa is of 3.3 GPa, its distal half has a higher Young’s modulus than its proximal half (close to 4 GPa and 3 GPa, respectively) [105]. This effect is probably due to the hollow medulla of the base, contrarily to the tip, which is compact [106]. Force is ultimately transmitted, since mechanoreceptors are located in the vibrissa follicle. Despite the Young’s modulus variation, the bending stiffness is higher in the base region, since it grows exponentially with the radius and deflections are smaller. In addition to the change of stiffness in longitudinal axis, the Young’s modulus decreases along the cross-section, towards the inner of the vibrissa [107].

A number of artificial whiskers have a straight cylindrical shape [85,96,108] (Figure 5E). Indeed, this particular shape increases the reachable space of the whisker compared to the typical natural shape [99]. Exploring the role of whisker morphology in active touch, the plastic whiskers arrays of the Shrewbot were made tapered and of different lengths [109], but elasticity does not vary along single whiskers. A way to tune bending elasticity along the shaft could be to vary the diameter, in order to protect the base from breaking, and to create a hollow inner towards the base, like natural whiskers. Artificial whiskers with an undulated surface in water have been implemented, the amplitude of their mechanical response being greater than that of vortex-induced vibrations, unlike a circular cylinder [49,110] (Figure 5F). 

### 2.2. Cantilevers with Domes

In nature, many cantilevers are found to be surrounded by a cupula, combining both other types of specialized mechanical sensory organs. Examples can be recognized in cupular organs and neuromasts, which are sensitive to flow velocity and acceleration [111,112].

#### Neuromasts

**Figure 6:**
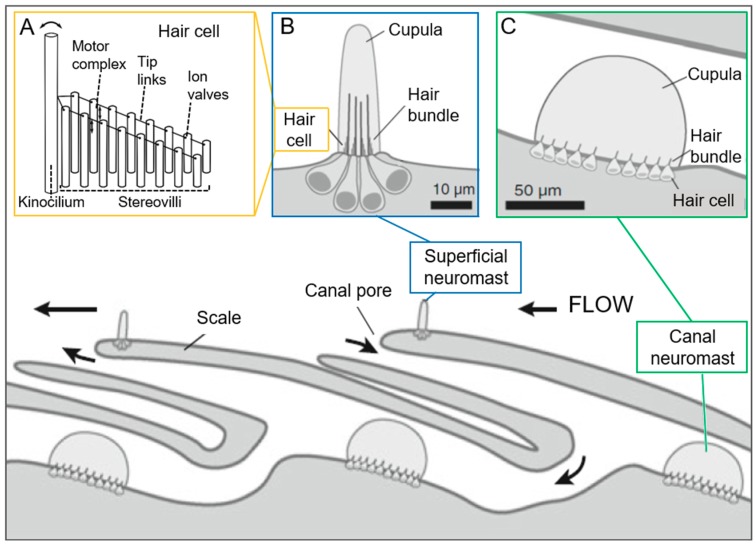
General structure and position of main components in the lateral line of fish. (**A**) hair cell of (**B**) superficial and (**C**) canal neuromasts ((**A**) reprinted from [74] by permission of John Wiley & Sons, Inc.; (**B**,**C**) and bottom reprinted by permission from Springer, [112]).

Neuromasts are found in the lateral line of the fish and deflect to water stimuli (Figure 6). They are classified in two categories according to whether their location is on the skin or underneath: superficial, which are sensitive to flow velocity, or canal neuromasts, which are sensitive to flow acceleration [111,113]. The hydrodynamic pressure threshold of canal neuromasts reaches 0.1–1 mPa [113]. Interestingly, in addition to the multiple behaviors enabled [114], like rheotaxis, orientation towards current [50], neuromasts of the cave fish permit three-dimensional (3D) hydrodynamic imaging, since they can even detect stationary objects [115]. Considering the particular use of flow sensing for fish, it is also referred to as “touch at a distance” [116,117]. 

Regarding neuromasts’ biomechanics, the outer cupula structure fulfills a double function: while it transmits the force perceived [74] as a mechanical band-pass filter [118], it helps avoid damage of the stereovilli and kinocilia embedded in it (Figure 6). Moreover, the presence of cupula enhances the organ’s force sensitivity by augmenting the drag. A force exerted from all directions provokes a cupula displacement, transmitted to the inner cantilevers. Cupula of superficial neuromasts have an oval shape, which enables discrimination of flow directionality, and a width of 10–60 µm, while canal neuromasts an hemispherical one with a diameter an order of magnitude higher [49,119]. 

Because of differences in location, dimensions, and interaction with the fluid, sensing mechanisms in superficial and canal neuromasts are different. These differences have been described through biomechanical models. In particular, in superficial neuromasts, the cupula is deflected by the flow behaving like an anchored beam, which is stiffer in the proximal region proportionally to the number of kinocilia, while compliant at the tip. According to this, superficial neuromasts have been modeled as two joined beams of different flexural stiffness, with a base acting as a pivot and a spring representing the coupling with the body [118,119]. Conversely, in canal neuromasts, the cupula slides along the sensory epithelium as a rigid body, and has therefore been modeled as a rigid hemisphere sliding over a frictionless plate, connected to a linear spring representing the coupling with the body [113,120]. According to mechanical models, in superficial neuromasts, the cupula size (radius and height) has a major influence on its mechanical behavior [118], while the cupula structure in canal neuromasts has a minor role [119]. In the range of sizes tested, the sensitivity’s maximal amplitude increases by increasing the cupula height or decreasing the cupula radius [118]. With a morphology similar to neuromasts, cupular organs are found in *Ciona intestinalis*, with a presumable mechanical sensing function even after brain removal [47]. An interesting morphological feature is that, contrarily to neuromasts, in these organs cantilevers are not found in the dome structure, macula [48], but on its top. In addition, cantilevers are surrounded by a “tunic-like material” [48], the cupula. Its shape is rather finger-like, merely enveloping and stiffening the cantilevers [47,48]. Regarding the macula structure, its function becomes clear through the function of cells it contains. They include sensory cells and supporting cells [48], giving mechanical stability to the structure above it receiving the signal.

Back in neuromasts, in the comparatively large cupula, the cantilever structures, cilia, are provided by the hair cells. Typically, cilia are organized in a graded stack (graded cilia principle) and connected between them. With this layout, an excitatory response occurs when deflected in direction of the longest cilium, the kinocilium, caused by the mechanical opening of the ion channels, and an inhibitory one in the opposite, since it does not permit opening of the channels. No bending along their shaft could be demonstrated [121], reminding trichobothria of arthropods. While canal neuromasts have a shorter cupula, compared to superficial neuromasts, they have a greater number of hair cells with kinocilia of greater length, suggesting the ability to perceive higher frequencies [50]. A theoretical model of the superficial neuromasts suggests increased mechanical sensitivity with longer kinocilia [118]. Additionally, in some species, such as the blind cave fish, cupular fibers with a supporting function for the cupula can be encountered [74,122]. They are thought to increase the bending stiffness of the cupula [118].

**Figure 7:**
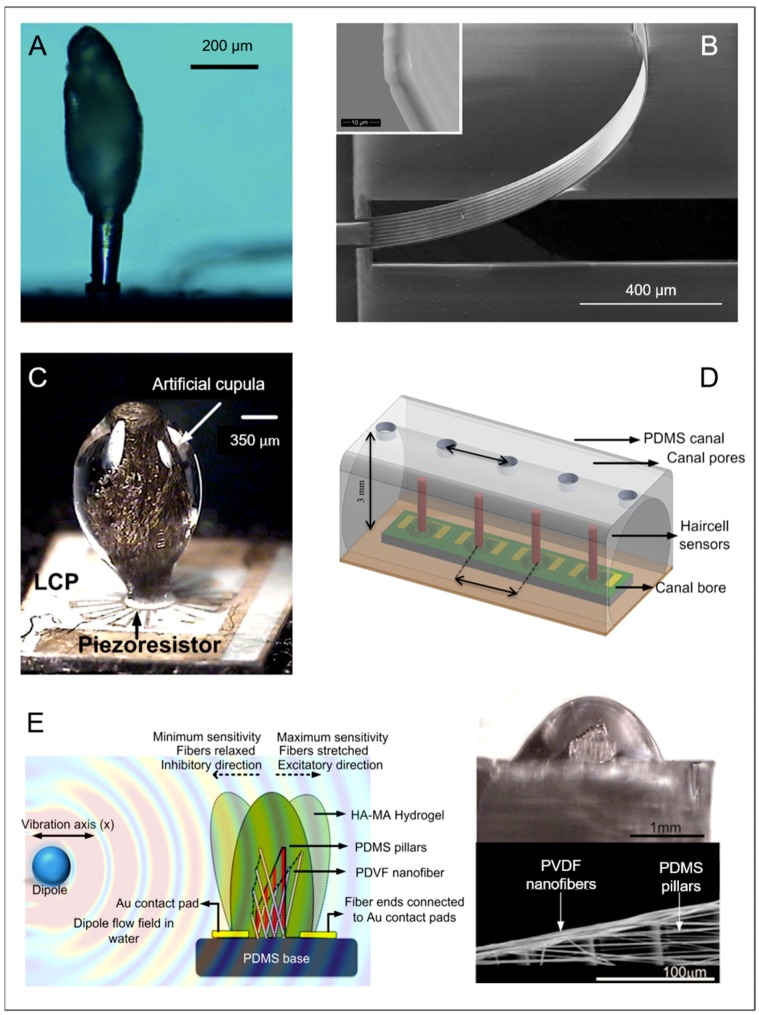
Artificial sensors with shape of cantilevers with domes. (**A**) Hydrogel cupula on hair sensor (reproduced from [36] with permission from The Royal Society of Chemistry). (**B**) Piezoresistive sensor with parylene coating (adapted from [123], Copyright 2012, with permission from Elsevier). (**C**) Hyaluronic acid methacrylic anhydride (HA-MA) based cupula (reproduced from [129], CC BY 4.0 [130]). (**D**) Artificial lateral line canal system (adapted from [126]. © IOP Publishing. Reproduced with permission. All rights reserved). (**E**) Piezoelectric sensor using the graded cilia principle (adapted from [124], CC BY 4.0 [130]). LCP: Liquid crystal polymer; PDMS: Polydimethylsiloxane; PVDF: Polyvinylidene fluoride.

For the design of artificial hair flow sensors, hair coating through the cupula could provide the necessary protection for sensor durability, whether for repeated cycles or for large forces applied. Applying the coating with a spherical shape assures displacement following stimuli from all directions. Secondly, the cupula transmits and amplifies force sensing by enhancing the drag. Indeed, artificial sensors using the principle of an additional cupula remark an augmented sensitivity, improving its deflection sensitivity up to 40 times [35] (Figure 7A). As seen earlier, in the cupular organs of *C. intestinalis,* hairs have a coating that adapts to their shape. Qualtieri et al. [123] used conformal parylene coating to make a cantilever sensor waterproof and tune its stiffness (Figure 7B). Having a dome support, like the macula, instead of a plane surface allows stimuli coming from all directions to be perceived. Displacement of the base region of the dome could also lead to a response, since its movement would also be transmitted to the upper structure. Having the neural part of the filter part directly in the dome, like the sensory cells in the macula, could potentially increase its sensitivity due to its major exposure to outer stimuli.

Numerous other sensors aim at mimicking neuromasts [114] (Figure 7), ranging from first attempts with soft/hard materials—for example, where SU-8 hairs are covered with a poly-(ethylene glycol) (PEG)-based hydrogel cupula with a low Young’s modulus of 8–10 kPa [51,122]—to artificial neuromasts, including soft natural polymers [124] (Figure 7C). In the latter solution, a polycarbonate hair is surrounded by an electrospun polycaprolactone (PCL) nanofibril scaffold and an artificial cupula made of a soft hyaluronic acid methacrylic anhydride (HA-MA) hydrogel. Ultimately, artificial lateral line canal systems have been engineered, mimicking canal neuromasts in their canal with pores that establish inner flow [125,126,127] (Figure 7D). Also, the cupula alone has been taken as an example, to build a microfluidic PDMS-based sensor using the capacitive principle [128].

The graded cilia principle mentioned previously is a method for achieving selectivity of flow direction. Indeed, bundles of flexible PDMS pillars of graded heights have already been used artificially, causing polyvinylidene fluoride (PVDF) nanofibers to stretch with a detection threshold as low as 8 µm/s, comparable with that of natural neuromasts [124] (Figure 7E). According to the size of each component, in this case those corresponding to the hair cells and those corresponding to the cupula, different functionalities can be achieved, such as fluid flow or acceleration. As mentioned previously and explained through a theoretical model [118], aspect ratio has a significant effect [36]. In case of soft materials used for the cupula of an artificial sensor, enforcing fibers might be necessary in order to maintain it upright [122].

### 2.3. Domes

Like cantilevers, cupulae or dome-shaped structures are also encountered separately as mechanical sensory organs. While most examples found are used in an aerial environment, alligators interestingly only react to water droplets when their face is half-submerged in water, not entirely in or out of water [44]. In this work, Soares reported the presence of integumentary sensory organs (ISOs) on their face and the inner part of their mouth, which are sensitive to surface waves. In crocodilians, ISOs are more sensitive than the human hand, which has higher indentation thresholds [131,132]. Another case, the intriguing star-nosed mole, *Condylura cristata*, is the fastest eating mammal with handling time reaching 120 ms [133] and has a motile star-like nose which only presents dome-shaped Eimer’s organs. Interestingly, Catania proposes a model for shape and texture coding, following brief compression of objects [40]. It is based on different deflection and consequent stimulation patterns of the nerve terminals positioned at the apex of Eimer’s organs. Finally, in plant organisms, which today are emerging as an effective model for soft biorobotics [10,134,135], the tactile blep is a dome-shaped structure on epidermal cells of tendrils, thought to be a specialized mechanical sensory organ, at which we will take a closer look [30,32]. On the other hand, the campaniform sensillum, which we will first discuss, is present in numerous insects and has a complex and variable shape, which includes a dome-shaped cap.

#### 2.3.1. Campaniform Sensillum

**Figure 8:**
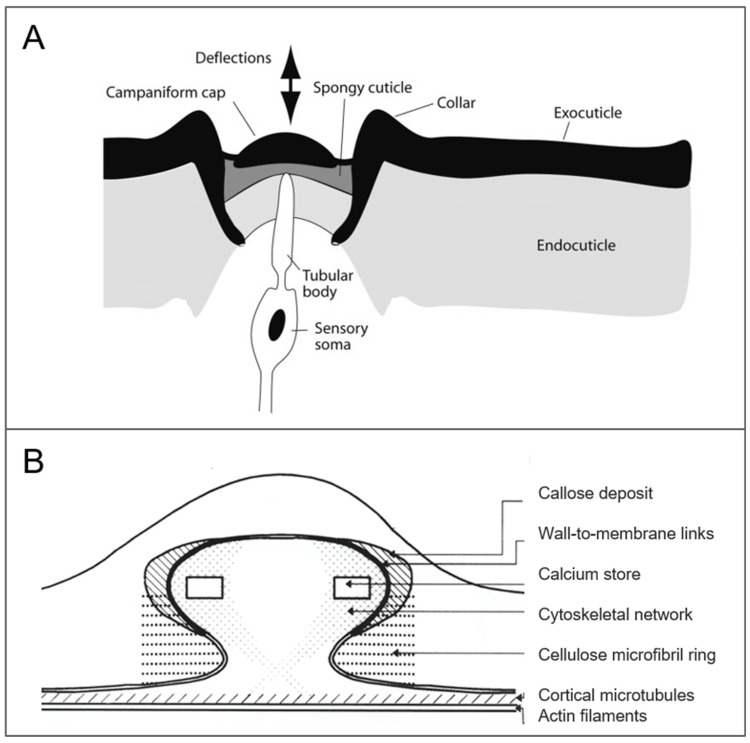
Examples of specialized mechanical sensory organs with a dome shape. (**A**) A diagram of the cross-section of a campaniform sensillum (adapted from [1] by permission of Oxford University Press). (**B**) Tactile blep architecture (reprinted by permission from Springer, [30]).

Campaniform sensilla are encountered in many insects, such as on the antennae [41] or trochanter [136] of the stick insect, or on the halteres of dipteran insects [33]. The campaniform sensillum has a round or more often oval outer shape, with a bell-like cap structure, and makes a swelling or depression in respect to the cuticle surface [42,137]. The cap structure is surrounded by a collar with a joint membrane, and has a spongy socket septum underneath [1] (Figure 8A). The campaniform sensillum of the cockroach legs, *Periplaneta americana*, which we will discuss next, provides information about cuticular strain generated through loading or postural changes. While it displays augmented responsiveness to self-induced forces [138], it also responds to external forces [43]. When located on legs, they are thought to be involved in flight inhibition, through activation upon contact of a substrate [42,139]. 

The 3D shape plays a decisive role in the behavior of the campaniform sensillum, with its cap structure transmitting the mechanical signal through its displacement. The campaniform sensillum responds to strain, preferably compression along its minor axis for oval-shaped ones, and consequent indentation of the cap cuticle, which stimulates the dendritic tip [43,137] (Figure 8A). The cap cuticle rotates the plane of movement by 90° [140]. 

What enables oval-shaped campaniform sensilla to discriminate directionality is the outer shape. Most groups on the legs are oriented in the same direction [42]. In a simplified way, a theory for the sensitivity dependence on the longitudinal length is exposed by means of a rubber-paper model by investigating distortion effects [42]. In this model, an essential assumption is the elasticity difference in the same structure. Indeed, materials in the complex structure play a decisive role. If the campaniform sensillum was built with an homogeneous material instead of heterogeneous ones, surrounding stress would cause it to move in the opposite direction [141].

In the artificial world, attempts to understand the natural mechanism have been made by modeling for artificial sensors designs. If needed according to the application, strain sensitivity and the consecutive indentation can be augmented through a dome shape compared to a flat structure within a depression [142,143]. The outer shape has an impact on strain amplification, augmenting it when it is elliptic, its major axis oriented vertically to the load [141]. Such a design could be used when maximal amplification is needed, while a change in shape and/or orientation permits to tailor the amplification, according to the input expected [141]. Considering the influence of materials, a careful selection and complex integration could be able to reverse an undesired mechanical behavior, as in the natural model.

#### 2.3.2. Tactile Blep

At first, the fact that plants are mechanosensitive might seem surprising. They respond to stimuli either in a slow way, through their developmental response, thigmomorphogenesis [144], or in a rapid way, which can be dependent (thigmotropic) or independent (thigmonastic) from the stimulus direction [31]. Mechanical stimuli affect the plant, which responds at a molecular, cellular, and macroscopic level [31,144,145,146,147,148,149]. Such responses occur in the aerial part, but also in roots [150]. An impressive example of fast response is given by the Venus’ flytrap, *Dionaea muscipula*, which snaps within about 100 ms when its hairs are touched twice consecutively [46,151,152]. *Mimosa pudica*, on the other hand, has a more delicate response, gently closing its leaves when touched [153,154].

On the tendrils of some plants, protruding hemispherical structures have been identified and studied to some extent, like the tactile bleps of *Bryonia dioica* Jacq. tendrils (Figure 8B). They are thought to act as specialized sensory organs for mechanoperception, despite lack of evidence, and to be more sensitive to shear than normal stimulation [30]. The dome shape structure of the tactile blep permits the displacement of the dome lower region to be larger when subjected to a shear force than to a normal force. The mechanical properties of its tissues vary greatly, in particular the elasticity, ranging from a fluid inner cytoplasm to a rigid cell wall [30]. Increasing the elasticity increases the structure displacement caused by the stimulus. The way, in particular the proportions, with which these materials are integrated and combined with each other, is thought to be another critical aspect.

**Figure 9:**
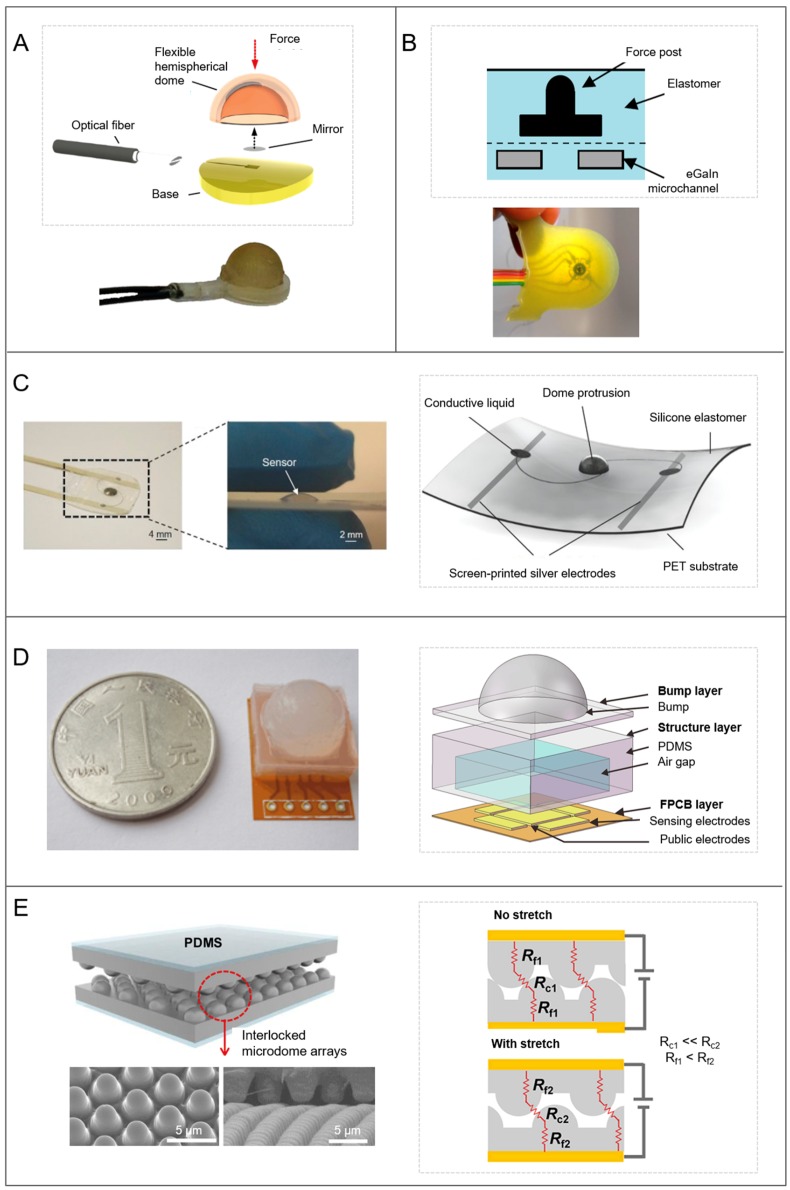
Artificial sensors integrating dome shapes. (**A**) Optical sensor (© 2014 IEEE. Reprinted, with permission, from [159]). (**B**) Piezoresistive sensor with liquid metals in an elastomeric layer (© 2013 IEEE. Reprinted, with permission, from [164]). (**C**) Piezoresistive sensor with liquid metals in a polyethylene terephthalate (PET) layer (reprinted from [165] by permission of John Wiley & Sons, Inc.). (**D**) Capacitive sensor with a multilayered dielectric on a flexible printed circuit board (FPCB) (reprinted by permission from Springer, [166]). (**E**) Piezoresistive sensor with bioinspired interlocking microstructured layers (adapted with permission from [171]. Copyright 2014 American Chemical Society). PDMS: Polydimethylsiloxane; R_c1_, R_c2_: Contact resistances; R_f1_, R_f2_: Film resistances.

Regarding the dome shape, numerous artificial sensors take advantage of it. For example, in order to perform slip and compression force detection in a robot hand during gripping of an object, a silicon rubber dome-shaped tactile sensor containing carbon microcoils (CMC) was employed in association to a force sensor [155]. A dome-shaped piezoelectric polymer (PVDF) was used to increase sensitivity in detecting a contact force [156]. Polydimethylsiloxane bumps covered with a conductive polymer (poly(3,4-ethylenedioxythiophene) polystyrene sulfonate, PEDOT:PSS) were proposed for high-performance resistive tactile sensing, and flexible pressure sensor arrays were fabricated in electronic skins [157]. In another example, dome-shaped piezoelectric transducers were obtained with the integration of stiff materials (aluminium nitride, AIN) on a softer one (polyimide) for the detection of dynamic contact forces [158]. Indeed, the dome shape is mostly used for fabricating sensors able to sense and discriminate multidirectional forces (Figure 9). They use different working principles in addition to different soft materials, like optical principle by means of fiber optics into an acrylonitrile butadiene styrene (ABS) polymeric structure [159] (Figure 9A); Hall effect obtained with a permanent magnet in a PDMS structure [160,161]; piezoelectricity by exploiting electroactive polymers (EAP) on a PDMS dome [162,163]; piezoresistivity by means of liquid metal components (eGaIn) embedded in Ecoflex elastomer layers [164,165] (Figure 9B,C); capacitive transduction associated to a multilayered dielectric formed by PDMS with an embedded air gap [166] (Figure 9D) or to a dielectric elastomer made with silicone emulsion [167]; or quantum tunneling in a fingertip three-axial sensing system, where quantum tunneling composites (QTC) were activated by the stress induced by a bump layer with a round mesa, used as a force transmission structure [168]. Moreover, designs inspired from mechanical properties of human skin were implemented. The TacTip is an optical-based sensor with a hemispherical shape, consisting of a hydrogel embedded camera, which can detect both the multidirectional force and shape of an object by monitoring the deformation of the skin through an embedded camera [169]. It has been developed in a family of sensors with different morphologies [170]. Moreover, piezoresistive sensors were fabricated, taking inspiration from interlocked epidermal/dermal ridges in the human skin [171,172] (Figure 9E). In particular, they were composed of multiwalled carbon nanotubes (MWCNT)/PDMS composites forming interlocked layers with different surface microstructures (e.g., dome, pyramid, and pillar). The best force sensitivities were obtained with microdome structures when inducing normal, tensile, and bending stresses.

Recently, our group has started investigating the mechanical behavior of the natural blep structure of *B. dioica* Jacq. tendrils, in order to shed light on the factors influencing it (i.e., geometry, material properties). Preliminary work by means of FEM modeling evaluates deformations in tactile bleps of different shapes, using values for the natural materials found in the literature [173]. In parallel, the model of an artificial capacitive sensor was simulated, with a simplified bilayer dome structure [174]. In this design, PDMS of different stiffnesses was considered for the dielectric dome layer and the outer dome skin. Even if these stiffnesses are different from the ones of the natural materials, the external skin has a higher Young’s modulus than the inner part (i.e., 5 MPa and 0.4–4 MPa, respectively). The results have demonstrated that, in addition to shape, material proportions play a decisive role. As an example, augmenting the proportion of the inner soft material leads to an augmented sensitivity to shear force. Additionally, while keeping the outer shape constant, normal and shear forces can be easily discriminated with particular configurations, obtained by tuning its biomechanics. This proof-of-concept demonstrates that making use of morphological computation on the mechanical part of the sensing filter can have a radical influence on the response given by the sensor.

## 3. Conclusions

Based on the examples of biological mechanical sensory organs presented (Table 1), mechanical principles have been identified. They contribute to their response by enabling or improving their functions, such as deflection and bending. Gaining such an overview of biomechanics in biological sensors permits us to identify key aspects that are transversal to different morphology types and environments (Table 2). Artificially, deliberate tailoring of the according structural features can bring new insights into their effects. Therefore, starting with the identification of relevant mechanical aspects, in the near future it could be possible to elaborate a toolbox of mechanical principles to be used during the design phase, in order to bring them in artificial sensors in a combined and methodical way. In the present day, artificial sensors have great capabilities of sensing a variety of stimuli. As stated initially, the types of stimuli needed to be measured include contact, pressure, force, strain, flow, vibration, acceleration, and directionality. However, different challenges are still to be met with a single sensor, namely, sensor softness, sensor robustness to repeated stimulations and durability, suppression of background noise, augmented sensitivity, light weight, mechanical multimodality (permitting to detect different mechanical stimuli), and in particular, simplification of the electrical layout and data processing.

First, some biological mechanical sensory organs are used for active touch, which means that organisms can actively respond to stimuli to gain supplementary information. To do this, orientation of the sensory organ or its supporting body part towards the stimuli is changed through active movement. Since it changes the way forces are experienced, orientation is a mechanical aspect of relevance. Second, specifications about shape play a fundamental role. Such specifications involve the presence of coating or not (e.g., through a cupula) and the surface aspect (e.g., the undulations present on harbor seal vibrissae, which have also been adopted artificially and reduce vortex-induced vibrations [49]). Length and diameter are also part of these specifications; for example, they both vary in flow sensing hairs with function of the boundary layer thickness in different media and at different flow speeds. The tuning of these aspects permits them to adapt to the environmental conditions expected, by either acting in synergy with them or protecting against them. As can be seen from the categorization, specific shapes seem to be predominant in a specific medium; for example, dome shapes are predominant in air, cantilevers with domes in water, while cantilevers appear in both air and water. Remarkable results have been obtained through biomimicry of the complex dome-cantilever structure of neuromasts [124]. Third, the distribution—for example, hair density—increases in areas of major importance or where multiple input types are provided—for example, in touch for exteroception or proprioception [175]. While increasing the density of the sensory organs might increase the resolution of the response, this is not always the case. Indeed, in nature, the concept of redundancy appears repeatedly and might be unnecessary for artificial designs. Fourth, the materials also play an important role. Some key aspects identified related to them, except their mechanical properties, are whether they are homo- or heterogeneous in structure, and the proportions between them. Generally speaking, in most cases studied, it seems that if the complexity of shape is increased—for instance, neuromasts with the complex shapes of their components—the complexity of the material structure is reduced and vice versa—for instance, spider tactile hairs with the regional heterogeneity along their shafts directly affecting the mechanical behavior. Fifth, micromechanics and regional particularities might radically change the system’s mechanical behavior. In the examples presented, they can regard the attachment site of the sensory organ with the rest of the body (e.g., the hair base), microstructures (e.g., microtrichs along flow sensing hairs), regional heterogeneities, whether of materials (e.g., through their elasticity) or of shape (e.g., through variation of diameter). They provide adaptation to a higher level, maximizing the system’s potential, making it more lightweight, functional, or robust.

Hence, different types of specialized sensory organs are distinguished—cantilevers and domes—but also their combination—cantilevers with domes. Investigating morphological computation in soft sensing, by providing a 3D structure and paying attention to the mechanical design in addition to the electrical design, is a path that is still poorly exploited. This analysis demonstrates with multiple examples that biomechanically tunable functionality and/or sensitivity is achieved in nature, and can also be achieved artificially with new sensors, such as the bilayer structure simplifying the natural model of the tactile blep [174]. 

The level of simplicity of principles used effectively in nature gives motivation for using them extensively in artificial sensors, where they are often overlooked. Comparing with artificial sensors, some of the described principles have been used (independently from bioinspiration), while other cases follow a specific natural model to different degrees, going towards biomechanical tuning. However, this approach can be further extended considerably in a structured manner, as a step following the trial-and-error approach.

Building soft sensors for traditional hard robots might bring an advantage, as seen previously for single sensors, but in soft robotics they are necessary in order to keep their compliant nature [176,177], by avoiding the use of rigid sensors. Another basic requirement in the real world is sensor robustness to repeated stimulations, as it is the case for natural organisms [176]. In many cases, they maintain sensory structures throughout their lives, which can span from days, like for the fly *Drosophila melanogaster* [178], to years for various mammals. 

Building on intrinsically soft materials or compliant mechanisms, soft robots are adding new robotic abilities that were unthinkable before (e.g., morphing and self-healing) [179], in addition to opening new possibilities for typical robotic tasks (e.g., grasping, dexterous manipulation, and locomotion) [176]. New levels of bioinspiration can be aimed for because of the intrinsic characteristics of the soft materials and smooth movements of the actuated structures. Therefore, soft robots represent ideal platforms for investigating the tight interplay between sensor and both the environment and own movements during typical tasks. Such experiments are also expected to demonstrate the importance of designing sensors in an integrated manner and not as discrete components.

To achieve the sensorization of soft robots, fabrication technologies play a decisive role. Today, we are assisting at a shift from two- to fully three-dimensional approaches, which are paving the way to the development of new sensors having smart 3D architectures at different scales, integrating materials with different mechanical properties. For example, the use of 3D printing for the fabrication of sensing elements embedding conductive components is growing fast [180,181,182], backed by the development of different printable materials [183,184] and printing techniques that allow combining materials with different mechanical properties in the same structure [185,186,187]. Furthermore, at the microscale, direct laser lithography (DLL) has been largely employed in biomimicry of 3D natural structures for surface functionalization [188,189,190,191], for structuring soft materials in three dimensions [192,193,194,195], also with a variable stiffness in the same structure [196,197], or for fabricating conductive 3D structures [198,199,200]. Nevertheless, many efforts are still necessary for the development of new materials that mimic the different properties of the natural ones, but that can still match fabrication requirements, especially for building heterogeneous mechanical structures.

In conclusion, biomechanical inspiration for sensors as a systemic methodology can be groundbreaking. Such a methodology might provide sensors of a great sensitivity, like those of canal neuromasts, but also simplify their electrical layout and data processing, like with the simplified bilayer of the tactile blep. Importantly, making a design toolbox available would be useful, not only for the design of sensors per se, but also for opening new possibilities in soft robotics, since optimization can only be obtained through an approach integrated with the rest of the body and the environment in which the robot is immersed. Sensorized soft robots themselves could provide useful platforms to shed light on the sensory mechanisms of the natural models. In addition, at the robotic system level, as soon as fabrication challenges are met, it will be thrilling to investigate in detail the advantages of deputing to the sensory biomechanical features a significant role in computing sensory information, with respect to the electronic and processing system. Finally, it is believed that morphological tunability could bring a new generation of sensors for both exteroception and proprioception, especially in soft robots.

## Figures and Tables

**Table:** 

Type	Mechanical Sensory Organ	Example Organism	Environment	Location/Distribution	Size
Domes	Tactile papilla	Chilean glory flower *Eccremocarpus scaber*	Air	Ventral and lateral side of branchlets	Base Ø 10 µm
	Tactile papilla	Snake* Rhinotyphlops*	Air	Rostrum, 250 papillae	Length 110 µm; Ø 26 µm
	Tactile blep *	Bryony *Bryonia dioica* Jacq.	Air	Similar density on upper and lower side of tendrils	Base Ø 4–5 µm
	Eimer’s organ *	Star-nosed mole *Condylura cristata*	Air/Soil	Star-like nose with 22 appendages, 25,000 organs	Ø 30–50 µm
	Campaniform sensillum	Stick insect *Carausius morosus*	Air	Antenna	Base Ø 5 µm
	Campaniform sensillum	Fly* Drosophila melanogaster*	Air	Halteres, 300 sensilla/haltere	Base Ø 10 µm
	Campaniform sensillum	Honey bee	Air	Head, elliptical form	Length 0.9 µm
	Campaniform sensillum *	Cockroach *Periplaneta americana*	Air	Leg in groups, semi-major axis in limb direction	Length 6–24 µm
	Integumentary sensory organ *	Alligator *Alligator mississippiensis*	Air/Water	Face and mouth inner	Base Ø 200 µm
Cantilevers	Hair sensillum *	Spider *Cupiennius salei*	Air	Legs (100 trichobothria per leg, 400 tactile hairs per mm^2^), joints	Length 0.1–3.2 mm; Base Ø 5–23 µm
	Hair sensillum	Honey bee	Air	Neck, 160–180 per hair plate, spacing 6–15 µm	Length 25–150 µm; Base Ø 2–5 µm
	Vibrissa *	Mouse	Air	Face	Macro- and microvibrissae
	Hair sensillum *	Venus *Dionaea muscipula*	Air	On each inner lobe of leaves, 3–5 sensilla	Length 2 mm; Base Ø 200 µm
	Hair cell	Jellyfish *Aglantha digitale*	Water	Velum and tentacle bases	Cilium length up to 30 µm, surrounded by graded microvilli
	Hair sensillum	Crayfish *Procambarus clarkii*	Water	Lateral antennular flagellum	Length 80–200 µm; Base Ø 5–15 µm
Cantilevers with Domes	Cupular organ *	Sea squirt* Ciona intestinalis*	Water	Siphons, 75–100 organs	Cupula length 250 µm; Macula base Ø 100 µm
	Cupular organ	Sea squirt* Corella eumyota*	Water	Branchial sac on atrial side, 34 organs	Cupula length 100–130 µm; Macula base Ø 80–100 µm
	Cupular strand	Sea squirt* Corella inflata*	Water	Dorsal fold of the branchial sac on atrial side, 1 organ	Length 7–8 mm; Width 20–30 µm
	Neuromast *	Fish *Astyanax fasciatus*	Water	Lateral line system, 'superficial' on skin surface, 'canal' in lateral line canals	Superficial: height 50–400 µm; Canal: order of magnitude higher

**Table:** 

Type	Mechanical Sensory Organ	Example Organism	Material	Exteroception	Proprioception	Detected Stimuli	Reference(s)
Domes	Tactile papilla	Chilean glory flower *Eccremocarpus scaber*	-	X		Touch **	[32]
	Tactile papilla	Snake *Rhinotyphlops*	-	X		Touch **	[19]
	Tactile blep *	Bryony *Bryonia dioica* Jacq.	Multiple (callose, cellulose, cell wall, cytoplasm)	X		Shear forces **	[30]
	Eimer's organ *	Star-nosed mole *Condylura cristata*	-	X		Touch	[40]
	Campaniform sensillum	Stick insect *Carausius morosus*	-	X	X	Shear forces for bending	[41]
	Campaniform sensillum	Fly *Drosophila melanogaster*	-	X	X	Strain	[26]
	Campaniform sensillum	Honey bee	Resilin, *E *= 1 MPa	X	X	Position/Inertia	[20,27]
	Campaniform sensillum *	Cockroach *Periplaneta americana*	Multiple	X	X	Strain	[42,43]
	Integumentary sensory organ *	Alligator *Alligator mississippiensis*	-	X		Flow/Touch	[44]
Cantilevers	Hair sensillum *	Spider *Cupiennius salei*	Cuticle, *E*= 18 GPa	X	X	Flow/Touch/Position	[28,45]
	Hair sensillum	Honey bee	Resilin (joint membrane), *E*= 1 MPa	X	X	Position/Inertia	[20,21,27]
	Vibrissa *	Mouse	-	X	X	Active touch/Self	[22]
	Hair sensillum *	Venus *Dionaea muscipula*	Multicellular, transversal sensory layer	X		Location	[23,31,46]
	Hair cell	Jellyfish *Aglantha digitale*	-	X		Flow	[24]
	Hair sensillum	Crayfish *Procambarus clarkii*	Torsional stiffness 10^−12^ Nm/°	X		Flow/Chemical	[25]
Cantilevers with Domes	Cupular organ *	Sea squirt *Ciona intestinalis*	Irregular folding of gelatinous proteinaceous cupula	X		Flow **	[47]
	Cupular organ	Sea squirt *Corella eumyota*	Irregular folding of gelatinous proteinaceous cupula	X		Flow **	[48]
	Cupular strand	Sea squirt *Corella inflata*	Finely fibrous proteinaceous cupula	X		Flow **	[48]
	Neuromast *	Fish *Astyanax fasciatus*	Gelatinous cupula, 10 kPa (superficial, blind cave fish)	X		Flow velocity/Acceleration	[49,50,51]

*: Example detailed in this paper; **: Hypothesized function; *E*: Young’s modulus.

**Table 2:** Mechanical aspects of specialized mechanical sensory organs, which are transversal to different morphology types and environments.

Mechanical Aspects	Details
1. Orientation	Of the sensory organ on the supporting body (e.g., for active touch)
2. Shape	Specific geometries according to the environment, length, diameter, coating (e.g., cupula), surface aspect (e.g., undulations)
3. Distribution	Often increased in areas of major importance or of multiple input types, redundancy
4. Materials	Mechanical characteristics, homo- or heterogeneity, proportions
5. Micromechanics	Shape and/or materials. Attachment site with the rest of the body (e.g., hair base), microstructures (e.g., microtrichs), shape (e.g., variation of diameter)
